# Gut-liver crosstalk in sepsis-induced liver injury

**DOI:** 10.1186/s13054-020-03327-1

**Published:** 2020-10-19

**Authors:** Jian Sun, Jingxiao Zhang, Xiangfeng Wang, Fuxi Ji, Claudio Ronco, Jiakun Tian, Yongjie Yin

**Affiliations:** 1 Department of Emergency and Critical Care Medicine, Second Hospital of Jilin University, Changchun, Jilin Province China; 2 International Renal Research Institute of Vicenza (IRRIV), Vicenza, Italy; 3 Department of Pharmacy, First Hospital of Jilin University, Changchun, Jilin Province China; 4 Department of Nephrology, Dialysis and Transplantation, San Bortolo Hospital, Vicenza, Italy

**Keywords:** Sepsis, Liver injury, Gut-liver crosstalk, Inflammation, Metabolism, Microbiota dysbiosis

## Abstract

Sepsis is characterized by a dysregulated immune response to infection leading to life-threatening organ dysfunction. Sepsis-induced liver injury is recognized as a powerful independent predictor of mortality in the intensive care unit. During systemic infections, the liver regulates immune defenses via bacterial clearance, production of acute-phase proteins (APPs) and cytokines, and metabolic adaptation to inflammation. Increased levels of inflammatory cytokines and impaired bacterial clearance and disrupted metabolic products can cause gut microbiota dysbiosis and disruption of the intestinal mucosal barrier. Changes in the gut microbiota play crucial roles in liver injury during sepsis. Bacterial translocation and resulting intestinal inflammation lead to a systemic inflammatory response and acute liver injury. The gut-liver crosstalk is a potential target for therapeutic interventions. This review analyzes the underlying mechanisms for the gut-liver crosstalk in sepsis-induced liver injury.

## Introduction

Sepsis is a life-threatening condition caused by dysregulated host response to infection [1]. Uncontrolled inflammation leads to loss of cellular and organ function, multiple organ dysfunction syndrome (MODS), and death [2]. Annually, more than 1.5 million patients suffer from sepsis in the USA, and the estimated global mortality rate is 25% [3, 4].

During systemic infections, the liver regulates immune defenses via bacterial clearance, production of acute-phase proteins (APPs) and cytokines, and metabolic adaptation to inflammation. Liver injury has a critical effect on the severity and outcome of sepsis. The incidence of liver failure in sepsis is lower than the incidence of failure of other organs because of the high regenerative capacity of the liver and its ability to withstand assaults [5]. However, liver dysfunction and failure are associated with grave complications in sepsis. Mortality rates among sepsis patients with liver dysfunction or failure range from 54 to 68%, which is higher than the mortality rates of sepsis patients with lung dysfunction or failure (the organ most commonly affected in sepsis) [6–10]. Although the pathophysiology of sepsis-induced liver injury is complex, the inflammatory response plays a major role in this process. Furthermore, mitochondrial and endoplasmic reticulum (ER) dysfunctions during the acute phase response elicited by systemic inflammation lead to liver failure in sepsis [11]. In critically ill patients, the clinical manifestations include hypoxic hepatitis, sepsis-induced cholestasis, and secondary sclerosing cholangitis [7].

**Fig. 1:**
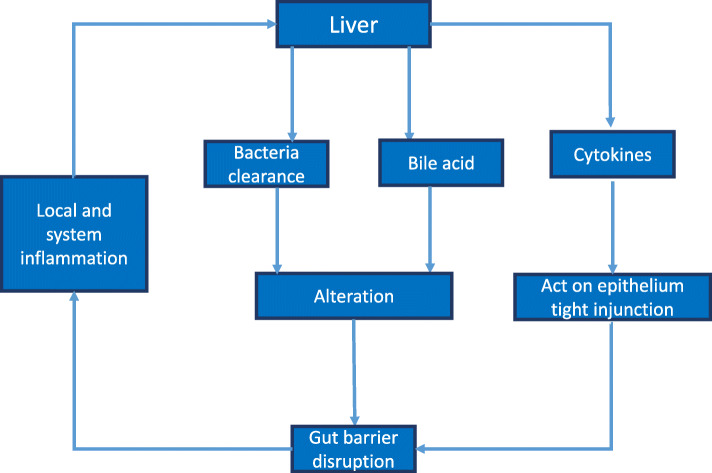
Effect of increased levels of inflammatory cytokines and decreased bacterial clearance in sepsis-induced liver injury on the gut

Intestinal barrier failure accompanied by systemic bacterial translocation was the predominant theory for the pathophysiology of MODS [12]. Bacterial pathogens frequently translocate from the gut lumen into the bloodstream and enter the liver via the portal vein [13]. Sepsis-induced epithelial apoptosis increases intestinal permeability (IP) and results in leakage of gut flora. Bacteria and their metabolites enter the lymphatic system through co-transport with chylomicrons, and dietary fat promotes the intestinal absorption of lipopolysaccharides (LPS) produced by intestinal bacteria [14]. These phenomena may exacerbate inflammation and lead to hepatic failure and death [15]. Gut dysbiosis and disruption of the intestinal barrier are caused by liver dysfunction, increased levels of inflammatory cytokines, impaired bacterial clearance, and metabolic disruption (Fig. 1). Changes in tight junctions and associated proteins due to liver dysfunction may increase gut permeability and damage distant organs. Therefore, a better understanding of the gut-liver crosstalk in sepsis-induced liver injury may help elucidate these complex disorders and form the basis for novel therapies.

## Effect of liver dysfunction on the gut

The liver is a major site for the regulation of immune and inflammatory responses [16]. During the acute phase of sepsis, inflammatory cytokines, mainly IL-6 and IL-1, trigger the production of acute-phase proteins (APPs) in hepatocytes. Activated macrophages or Kupffer cells (KCs) and natural killer T (NKT) cells produce inflammatory mediators, including interferons (IFNs), IL-1β, IL-8, and tumor necrosis factor (TNF). Neutrophils and monocytes accumulate in the liver and contribute to antimicrobial defense (via neutrophil extracellular trap [NET] formation or phagocytosis [17]) and immune-mediated hepatocyte injury (through TNF-induced apoptosis) [7, 16].

During sepsis, liver injury varies from active hepatocellular dysfunction to life-threatening fulminant hepatic failure and induces severe and systemic detrimental inflammatory responses in other organs [18]. Liver dysfunction is accompanied by an increase in inflammatory cytokines and impairment of bacterial clearance and excretion of bile acids, which may cause gut microbiota dysbiosis and disruption of the intestinal barrier.

### Effect of cytokines on the gut barrier during sepsis

**Fig. 2:**
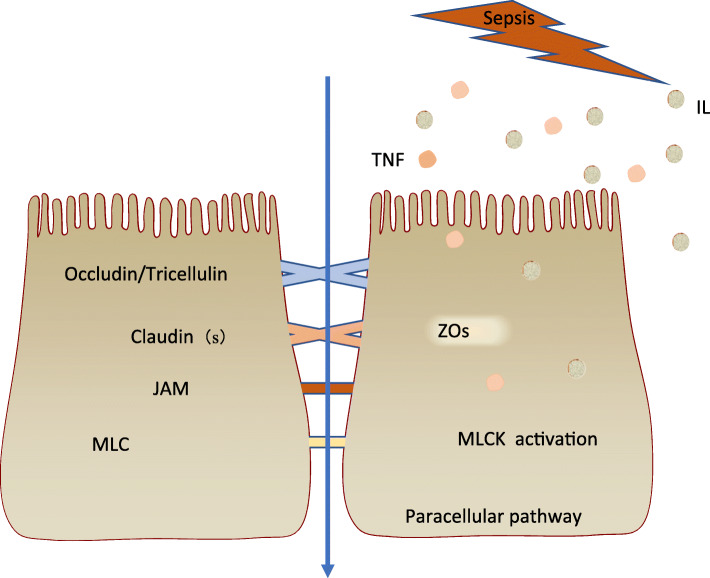
Effect of cytokines on the gut barrier during sepsis. During sepsis, increased levels of cytokines can modulate gut permeability by regulating the protein expression of claudins, JAM-A, occludin, and ZO-1. The phosphorylation of myosin regulatory light chain by myosin light chain kinase (MLCK) can increase paracellular permeability and result in contraction or opening of the apical tight junctions

The single layer of epithelial cells in the intestinal tract acts as a selective barrier, allowing paracellular transport of water, solutes, and immune factors while preventing the transfer of potentially harmful pathogens, toxins, and antigens from the lumen to the circulation and mesenteric lymph [19–21]. Tight junctions maintain gut barrier function [22] and are linked to the intracellular cytoskeleton by several families of intramembrane proteins (claudin, occludin, tricellulin, junctional adhesion molecule [JAM]) and intracellular connector proteins (zonula occludens [ZO], myosin light chain), which help bridge the intercellular space [23]. During sepsis, increased levels of cytokines can modulate gut permeability by regulating the protein expression of claudin 2, claudin 5, JAM-A, occludin, and ZO-1 [24]. Myosin light chain kinase (MLCK) activation can increase the levels of interleukin (IL)-6, tumor necrosis factor α (TNF-α), and IL-1β. The phosphorylation of myosin regulatory light chain by MLCK can increase paracellular permeability and result in contraction or opening of the apical tight junctions [23, 25], leading to the activation of MLCK in a feed-forward mechanism, in part via altered ZO-1 and occludin [26] (Fig. 2). Increased intestinal permeability leads to increased systemic inflammation via a positive feedback loop [27].

MLCK inhibition improved survival in a mouse model of sepsis [28] and improved barrier function and tight junction protein assembly in a murine model of burn injury [29]. These results suggest that MLCK inhibition is a potential treatment modality in sepsis and a critical target for reversing intestinal epithelial barrier disruption after severe burn injury.

Toll-like receptor 4 (TLR4) regulates the proliferation and apoptotic death of intestinal stem cells [30]. The increase in the level of cytokines during sepsis inhibits gut cell regeneration and stimulates apoptosis in a TLR4-dependent manner [31]. On a cellular level, the proliferation of crypt cells is decreased, while crypt and villus apoptosis are simultaneously increased [32]. Sepsis decreases intestinal cell proliferation, increases intestinal epithelial apoptosis, and reduces villus height [33]. Decreased villus height is associated with increased intestinal permeability, increased apoptosis of intestinal epithelial cells (IECs), and decreased survival [34]. Moreover, cytokines induce alterations in the mucus layer (reduced thickness, diminished luminal coverage, and poor adherence) and gut barrier function [35]. An increase in the level of inflammatory cytokines results in intestinal apoptosis and hyperpermeability, bacterial translocation, and expansion of the inflammatory responses [36]. Furthermore, increased systemic inflammation leads to liver injury.

### The role of bacterial clearance and gut dysbiosis in the gut-liver axis

The liver is an essential site for bacterial phagocytosis and clearance from the bloodstream [37]. KCs, liver sinusoidal endothelial cells (LSECs), and stellate cells are the first line of defense against blood-borne bacteria.

Phagocytic KCs clear bacteria and bacterial metabolic products by pattern-recognition receptors [38] KCs secrete chemokines, leading to neutrophil migration and accumulation in liver sinusoids. Neutrophils and platelets interact with each other and capture and kill bacteria via neutrophil extracellular traps [39]. Liver B cells also phagocytose bacteria [40]. Hepatocytes phagocytose pathogens that cross the sinusoidal cell layer [41]. Hepatocytes use lipoproteins to clear LPS. LPS-binding protein transfers LPS to CD14 and facilitates the interaction between LPS and TLR4 on the surface of phagocytes to remove LPS and initiate the pro-inflammatory cascade [42]. The increased level of inflammatory cytokines results in gut barrier dysfunction.

The gut and the associated local immune system work together to prevent translocation of intestinal bacteria into the portal vein. After the gut intestinal epithelial barrier, the liver constitutes the second line of defense in eliminating invading bacteria and bacterial products, inhibiting a spread of bacteria into the body [13, 37]. During the liver injury, the impairment of the reticuloendothelial system, ability of neutrophils to phagocytose and kill bacteria, complement production, and antigen presentation (downregulation of monocyte human leukocyte antigen DR expression) impair bacterial clearance [43]. Accordingly, patients with liver cirrhosis have an increased risk of bacterial infections, which is often due to bacterial translocation from the intestine [44, 45].

The altered gut microflora plays an important role in the gut-liver axis. Gut dysbiosis, may damage the gut barrier and increase permeability [46]. Schnabl has shown that gut dysbiosis seems to participate in the disruption of intestinal epithelial tight junctions and imbalance in the proliferation and apoptosis of IECs [13]. Recent research has found that gut microbial dysbiosis leads to serious liver injury in mice after cecal ligation and puncture [47]. Several mechanisms may answer this phenotype. First, liver anatomy allows close interaction with the gut, where nutrients and microbial products contribute to the maintenance of healthy metabolism and liver homeostasis. Second, through the portal circulation, intestinal products are transported to the liver [48]. Therefore, gut dysbiosis directly aggravates sepsis-induced liver injury.

Another clinically relevant consequence of gut dysbiosis is the induction of inflammation with hemodynamic derangement, which is caused by the translocation of intestinal bacteria to the peritoneal cavity and the systemic circulation [49].

In summary, the alteration of gut microbial communities and the translocation of bacteria and toxins increase the systemic inflammatory response and contribute to multiple organ failure and death [50]. The interplay between the gut and the liver may amplify the inflammatory response and contribute to the development of sepsis and MODS.

### Role of bile acids in the gut-liver axis

**Fig. 3:**
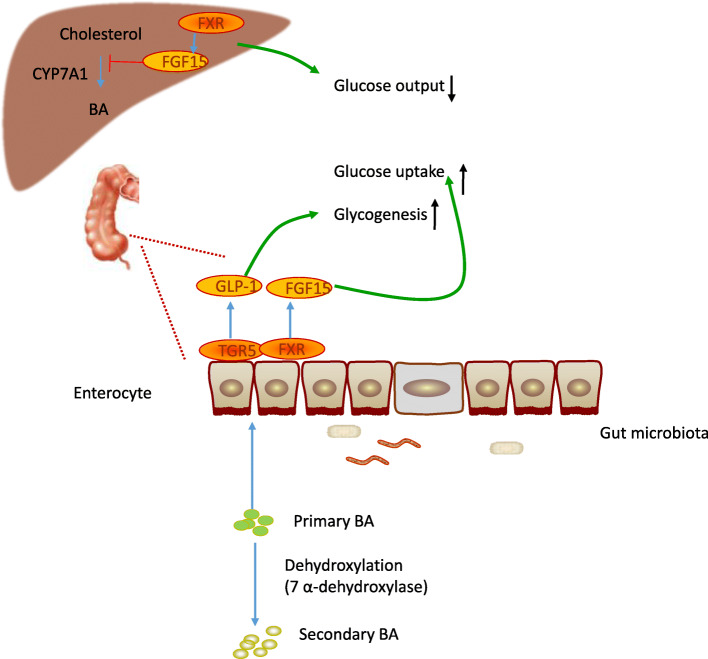
The role of bile acids in the gut-liver axis. During sepsis, the liver receives microbial input and influences intestinal microbes via bile acids. The gut microbiota can contribute to the pathogenesis of sepsis-induced liver injury by altering bile acids metabolism and its signaling pathways

The liver is an endocrine gland that secretes bile acids (BAs) into the intestine and regulates the composition of the intestinal flora [51]. BAs are synthesized from cholesterol in the liver and chemically modified by the gut microbiota in the distal small intestine and colon (Fig. 3) [52]. The majority (95%) of BAs are reabsorbed in the distal ileum and return to the liver through the portal circulation in a process known as the enterohepatic cycle [53].

During liver dysfunction, the increased BA levels activate inflammatory and oxidative stress and result in apoptosis or necrosis, fibrosis, and cirrhosis [54, 55]. BAs are key signaling molecules [56]. The farnesoid X receptor (FXR) and G protein-coupled receptors such as TGR5 mediate the regulatory actions of BA [57]. The microbiota modify signaling through receptors via its metabolism of BAs [58]. BAs activate FXR in the intestine and induce the expression of fibroblast growth factor 15 (FGF15). FGF15 decreases BA levels through a gut-microbiota-liver feedback loop by suppressing the expression of cholesterol 7α-hydroxylase (CYP7A1) in the liver—the rate-limiting step in BA synthesis [52]. In addition, FXR activation controls hepatic de novo lipogenesis, triglyceride (TG) transport by very-low-density lipoprotein (VLDL), and gluconeogenesis [59]. Secondary BAs activate TGR5, which improves glucose tolerance and liver function by inducing the release of intestinal glucagon-like peptide-1 (GLP-1) from intestinal enteroendocrine L cells [60]. Therefore, BAs regulate lipid and glucose homeostasis in the liver [59].

In the intestine, bacteria metabolize BAs to hydrophobic species through 7α-dehydroxylation and/or deconjugation of hydrophilic groups, resulting in the formation of the secondary BAs deoxycholic acid and lithocholic acid [61]. Moreover, BAs have bactericidal activity and reduce the intestinal permeability to endotoxins [62]. However, increased BA deconjugation reduces the bactericidal properties of the bile, causing the growth of bacteria that promote further BA deconjugation, and ultimately, bacterial translocation and endotoxemia in homeostasis conditions [62]. Higher levels of unconjugated primary BAs in the stool are correlated with intestinal dysbiosis [63]. Gut dysbiosis can disrupt intestinal epithelial tight junctions and cause an imbalance between the proliferation and apoptosis of IECs [13].

In summary, the liver may play a crucial regulatory role in controlling microbial populations by receiving microbial input and influencing intestinal microbes via BAs [64]. The gut microbiota contributes to the pathogenesis of sepsis-induced liver injury by altering BA metabolism and its signaling pathways. In the gut-liver axis, changes in BA metabolism cause an imbalance of the intestinal microflora and liver inflammation [65]. The interplay of BA between gut microbiota and the liver may promote organ inflammation and injury during sepsis.

## Effect of sepsis-related gut injury on the liver

The gut contains 70–80% of the body’s immune cells and more than 100 trillion microorganisms [66]. Inflammation and hypoperfusion play an important role in the pathophysiology of gut injury in sepsis patients [67]. Sepsis increases apoptosis, decreases proliferation, and reduces migration of the epithelium. Furthermore, changes in tight junctions result in intestinal hyperpermeability and gut barrier dysfunction. Several other changes occur in the gut physiology in septic patients, due to extrinsic factors (antibiotics and parenteral nutrition) and intrinsic factors (systemic inflammation). These changes, in turn, influence the composition of the enteric flora [68]. Damage to the gut barrier causes the translocation of bacteria and toxins from the intestinal lumen to the mesenteric lymph nodes and systemic circulation. Bacterial translocation may increase systemic inflammation, leading to multiple organ failure and death [50].

Monocytes/macrophages play a vital role in the initiation or progression of inflammatory diseases [69]. KCs are involved in the regeneration of liver homeostasis by serving as gatekeepers for the clearance of pathogens [70]. KCs are crucial for host defense against bacteremia by clearing 70–80% of bloodstream bacteria [71].

After gut injury, bacteria and their products enter the liver from the gastrointestinal tract via the portal circulation and lymphatic system. Evidence suggests that the principal route of bacterial translocation is the lymphatic system.

Pathogen-associated molecular patterns (PAMPs) are exposed to the liver and recognized by pattern-recognition receptors (PRRs), which belong to the TLR and NOD-like receptor families [72]. PAMPs bind to KCs or stellate cells and induce the transcriptional upregulation of pro-inflammatory genes coding for cytokines such as IL6, IL-1β, and TNFα. The production of APPs is initiated when the liver switches from tolerogenic towards immunogenic responses [73]. Many of the APPs contribute to systemic immunity by acting as opsonins, activating neutrophils or macrophages via PRRs, and antimicrobial functions of the complement system [74–76].

Since the polarization of KCs plays an important role in liver injury, modulating KC function is essential for protection against liver injury during sepsis [77]. KCs are polarized into distinct phenotypes, M1 (proinflammatory) and M2 (alternative), depending on the local microenvironment. During sepsis, as the gut barrier function is impaired, increased bacterial translocation and inflammation change the immune microenvironment, and KCs are polarized into an M1 phenotype. Studies showed that enhancing M2 polarization in macrophages could protect the liver against injury [78, 79]. However, further investigation is needed to identify the signaling pathways involved in inhibiting the M1 phenotype in macrophages.

Bacterial metabolites such as short-chain fatty acids (SCFAs) maintain immune homeostasis [80]. SCFAs enhance the intestinal barrier function and promote the maturation of regulatory T (Treg) cells [80]. A recent study found that SCFA-producing bacteria improved colonization resistance against enteric pathogens in animals and humans. Notwithstanding, additional controlled studies are required to assess whether dietary fiber can modulate the gut microbiome and benefit patients in the intensive care unit [81].

The liver immune response contributes to bacterial clearance but may lead to organ damage due to an overwhelming systemic inflammatory response. Mitochondrial dysfunction and ER stress-related inflammation during the acute phase response culminate in liver failure.

## Future prospects

As the gut plays an important role in the progression of sepsis-induced liver injury, targeting microbiota can have preventive and therapeutic potential in liver dysfunction. Some observations suggest that microbiome depletion is related to the association between antibiotic exposure and subsequent sepsis [82]. Moreover, modulation in target microbiota in at-risk patients before sepsis onset may decrease the incidence of sepsis. Treatments with fecal microbiota transplantation and microbiota-targeted metabolites, including butyrate or other SCFAs, may use to treat dysbiosis during sepsis. But the benefits of fecal microbiota transplantation should be balanced with the associated risks such as infectious complications [83]. Based on these findings, probiotics and synbiotic preparations can decrease the incidence of infections in the ICU [84–87].

## Conclusions

A large amount of literature supporting the possibility of the strong interplay between gut and liver in sepsis has been reviewed. The translocation of bacteria or metabolites and the resulting intestinal inflammation lead to liver dysfunction. Furthermore, sepsis-induced liver injury can promote gut dysbiosis and increase intestinal permeability. Increased permeability of the mesenteric vasculature causes intestinal edema and gut barrier dysfunction, leading to systemic inflammation and acute liver injury. Long-term inflammation leads to devastating consequences. Further understanding of the gut-liver crosstalk may provide insights on homeostatic regulation in sepsis and help develop effective therapies to prevent sepsis-induced liver damage.

## Data Availability

Not applicable.

## Abbreviations

APP: Acute-phase proteins
BAs: Bile acids
DAMPs: Damage-associated molecular patterns
ER: Endoplasmic reticulum
FGF15: Fibroblast growth factor 15
FMT: Fecal microbiota transplantation
FXR: Farnesoid X receptor
GLP-1: Glucagon-like peptide-1
IL: Interleukin
IP: Intestinal permeability
JAM: Junctional adhesion molecule
LSECs: Liver sinusoidal endothelial cells
MLCK: Myosin light chain kinase
MODS: Multiple organ dysfunction syndrome
NET: Neutrophil extracellular trap
PAMP: Pathogen-associated molecular patterns
PRR: Pattern-recognition receptors
SCFAs: Short-chain fatty acids
TLR4: Toll-like receptor
TNF-α: Tumor necrosis factor α
VLDL: Very-low-density lipoprotein
ZO: Zonula occludens
